# Mediating effect of self-disclosure between intolerance of uncertainty and existential distress in young and middle-aged patients undergoing maintenance hemodialysis

**DOI:** 10.3389/fpsyg.2026.1730213

**Published:** 2026-03-11

**Authors:** Hong Pan, Xisheng Xie, Mi Li

**Affiliations:** Department of Nephrology, Beijing Anzhen Nanchong Hospital of Capital Medical University & Nanchong Central Hospital, Nanchong, Sichuan, China

**Keywords:** existential distress, intolerance of uncertainty, maintenance hemodialysis, mediating effect, self-disclosure, young and middle-aged

## Abstract

**Background:**

The proportion of young and middle-aged patients undergoing maintenance hemodialysis (MHD) has increased in recent years. Due to the physical and psychological impacts of the disease, MHD patients are prone to existential distress, which can lead to anxiety, depression, and other psychological issues. This not only severely impairs their quality of life but also imposes a heavy burden on families and society.

**Objective:**

To explore the mediating role of self-disclosure between intolerance of uncertainty and existential distress in young and middle-aged MHD patients, and to provide intervention targets for reducing existential distress in this population.

**Methods:**

From January 2024 to October 2024, 342 MHD patients treated in the Hemodialysis Center of a tertiary grade A hospital in Nanchong were selected using convenience sampling. A general information questionnaire, the Intolerance of Uncertainty Scale (IUS), the Self-Disclosure Index (SDI), and the Existential Distress Scale (EDS) were used for data collection.

**Results:**

The scores of self-disclosure, intolerance of uncertainty, and existential distress in young and middle-aged MHD patients were (38.08 ± 5.97), (45.44 ± 6.10), and (34.39 ± 7.72), respectively. Significant differences in intolerance of uncertainty scores were observed among patients with different genders, age groups, dialysis durations, marital statuses, educational levels, and employment statuses (all *p* < 0.05). Similarly, self-disclosure scores differed significantly by gender, age group, dialysis duration, educational level, and employment status (all *p* < 0.05), and existential distress scores varied significantly by gender, age group, dialysis duration, marital status, educational level, and employment status (all *p* < 0.05). Intolerance of uncertainty was negatively correlated with self-disclosure (*r* = −0.766, *p* < 0.01), positively correlated with existential distress (*r* = 0.819, *p* < 0.01), and self-disclosure was negatively correlated with existential distress (*r* = −0.817, *p* < 0.01). Self-disclosure played a partial mediating role between intolerance of uncertainty and existential distress, accounting for 43.2% of the total effect.

**Conclusion:**

Intolerance of uncertainty can directly affect existential distress and indirectly influence it through the mediating role of self-disclosure. Medical staff should not only take measures to reduce patients’ intolerance of uncertainty but also promote their active self-disclosure to further lower the level of existential distress.

## 1 Introduction

Chronic Kidney Disease (CKD) is a chronic condition characterized by irreversible damage to kidney structure and function caused by multiple factors. It has an insidious onset, high prevalence, low awareness rate, and poor prognosis, making it a major global public health challenge (United States Renal Data System, 2023; Jha et al., 2013). Terminology for kidney function and disease is reported in accordance with the nomenclature established by Levey et al. (2019). When CKD progresses to the end stage (End-Stage Renal Disease, ESKD), patients rely on renal replacement therapy to sustain life. Among various therapeutic options, Maintenance Hemodialysis (MHD) has become the most widely used modality for ESKD patients worldwide due to its mature technology and proven efficacy (Liyanage et al., 2015; Ho et al., 2022). As the country with the largest number of MHD patients globally, China had approximately 749,500 patients receiving MHD treatment by December 2021. Notably, the age of MHD patients shows a significant younger trend, with the proportion of young and middle-aged patients increasing year by year. This trend toward a younger dialysis population may be attributed to a combination of factors, including shifts in epidemiology (such as the rising prevalence of diabetes and hypertension in younger adults), lifestyle factors, improved long-term survival allowing more patients to reach dialysis, and potentially earlier diagnosis and referral (Mai et al., 2023). This not only imposes a heavy burden on individual patients and their families but also exerts a substantial impact on the social labor structure and the allocation of public health resources (Kuma and Kato, 2022; Song et al., 2024; National Center for Quality Control of Nephrology, 2021).

Young and middle-aged individuals are in a critical stage of life development, serving as both the economic backbone of families and the core force in social construction. However, they endure physical suffering from disease-related complications such as fatigue, severe anemia, cardiovascular issues, and mineral bone disorders. They also face significant psychological difficulties, including anxiety, depression, disruption of family and social roles, and employment challenges due to the demanding treatment schedule. These multiple burdens severely impair their physical and mental health, leading to a significant decline in quality of life (He et al., 2022). Among these challenges, existential distress—a core psychological dilemma—refers to psychological turmoil arising from the loss of existential value and sense of meaning. Prolonged exposure to this state can cause patients to develop feelings of helplessness and frustration, even losing confidence in treatment and life. In extreme cases, it may contribute to suicidal ideation, as evidenced by studies highlighting the severe psychological burden in dialysis populations (Miyata et al., 2018; Wang X. et al., 2024; Wang Y. Y. et al., 2024), making it a key psychological factor affecting treatment adherence and survival outcomes in young and middle-aged MHD patients.

Among the numerous factors influencing existential distress, intolerance of uncertainty is a crucial trait-like variable. It refers to a persistent state of anxiety exhibited by individuals when facing uncertain events (e.g., disease progression, fluctuations in treatment efficacy). This anxiety directly exacerbates emotional disturbance and further amplifies the experience of existential distress (Cleveland et al., 2022). In contrast, self-disclosure—an important psychological adjustment behavior—involves individuals actively expressing their inner thoughts and emotions through verbal communication, written records, or other means after experiencing negative events. Existing studies have confirmed that self-disclosure can effectively alleviate negative psychological states (Pathmalingam et al., 2023). The Social Penetration Theory further points out that self-disclosure is a fundamental form of social exchange: when individuals confront painful experiences and future uncertainties, they seek external support and understanding through active self-disclosure, thereby reducing psychological burdens (Yang et al., 2023). Based on this theory and existing research clues, we reasonably hypothesize that intolerance of uncertainty may indirectly affect existential distress by influencing self-disclosure. However, the specific pathway and mechanism of interaction among these three variables remain unclear. This research gap hinders the development of targeted clinical interventions, making it difficult to effectively alleviate existential distress in young and middle-aged MHD patients.

Existing studies have confirmed that the level of existential distress in young and middle-aged MHD patients is significantly higher than that in dialysis patients of other age groups, and intolerance of uncertainty is prevalent in this population. Nevertheless, empirical research on “whether self-disclosure plays a mediating role between these two factors” remains scarce. Clarifying the relationship among these three variables will not only enrich the theoretical system of psychological intervention for patients with chronic kidney disease but also provide specific intervention targets for clinical practice. For instance, if the mediating role of self-disclosure is confirmed, medical staff can enhance patients’ self-disclosure ability to break the vicious cycle of “intolerance of uncertainty → existential distress.”

Therefore, this study focuses on young and middle-aged MHD patients. It aims to systematically analyze the current status and influencing factors of existential distress, with a particular focus on exploring the mediating role of self-disclosure between intolerance of uncertainty and existential distress. The ultimate goal is to clarify the mechanism of interaction among these three variables, thereby providing a scientific basis for medical staff to collaborate with families in developing individualized psychological intervention programs, alleviating patients’ existential distress, and improving their quality of life.

## 2 Materials and methods

### 2.1 Study participants

From January 2024 to October 2024, MHD patients in the Hemodialysis Center of Nanchong Central Hospital (Nanchong Hospital of Beijing Anzhen Hospital Affiliated to Capital Medical University) were selected by convenience sampling. Inclusion Criteria: (1) Diagnosed with CKD requiring MHD according to the 2024 KDIGO Clinical Practice Guideline for the Evaluation and Management of Chronic Kidney Disease (Zhang et al., 2024); (2) Aged 18 to <60 years; (3) Hemodialysis duration >3 months(A dialysis vintage of ≥3 months was selected to ensure that participants had passed the initial acute adjustment phase and had established a relatively stable treatment routine, thereby providing more reliable responses regarding their chronic psychological experiences.); (4) Clear consciousness and ability to communicate normally. Exclusion Criteria: (1) Unstable condition with potential acute deterioration; (2) Complicated with other malignant tumors; (3) Receiving psychological treatment. This study was approved by the Medical Ethics Committee of Nanchong Central Hospital (Approval No.: 2020(044)), and all participants provided written informed consent. Sample Size Calculation: The sample size was calculated using the formula *n* = (μ*α*/2 × *σ*/*δ*)^2^ (Li and Liu, 2018).where *α* = 0.05, the allowable error δ = 2.0, and the standard deviation of existential distress was 10.78 based on previous studies (Jha et al., 2013). The initial sample size was 198, and a 20% attrition rate was added, resulting in a final estimated sample size of 238. However, during the data collection period, we were able to recruit a larger sample (*N* = 342) to enhance the statistical power and robustness of the mediation analysis.

### 2.2 Study instruments

#### 2.2.1 General information questionnaire

Developed by the research team based on the study objectives and literature review, which included variables commonly assessed in studies of chronic illness and quality of life, such as age, gender, marital status, educational level, monthly family income, employment status, dialysis duration, and dialysis frequency (Hu et al., 2024).

#### 2.2.2 Intolerance of Uncertainty Scale (IUS)

Developed by Carleton et al. (2007), this scale assesses patients’ intolerance of uncertainty regarding disease and life. It consists of 12 items across 2 dimensions: prospective anxiety (7 items) and inhibitory anxiety (5 items). Each item is scored on a 5-point Likert scale (1 = “Strongly Disagree” to 5 = “Strongly Agree”), with a total score ranging from 12 to 60. Higher scores indicate greater intolerance of uncertainty. The scale has been validated in colorectal cancer patients, showing good reliability and validity (Cronbach’s *α* = 0.879). In this study, the Cronbach’s α coefficient was 0.886.

#### 2.2.3 Self-Disclosure Index (SDI)

Developed by Kahn and Hessling (2001) and localized for the Chinese population by Li (2009), this scale measures patients’ ability to express disease-related worries and feelings to others. It includes 12 items in a single dimension, each scored on a 5-point Likert scale (1 = “Strongly Disagree” to 5 = “Strongly Agree”). The total score ranges from 12 to 60, with higher scores indicating better self-disclosure. Score ranges are defined as follows: low self-disclosure (12–29), moderate self-disclosure (30–44), and high self-disclosure (45–60). In this study, the Cronbach’s *α* coefficient was 0.812.

#### 2.2.4 Existential Distress Scale (EDS)

Developed by Yu (2024) specifically for hemodialysis patients, this scale evaluates patients’ existential distress. It includes 14 items across 4 dimensions: loss of control (3 items), loneliness (4 items), guilt (3 items), and meaninglessness and valuelessness (4 items). Each item is scored on a 5-point Likert scale (0 = “No Distress” to 4 = “Extreme Distress”), with a total score ranging from 0 to 56. Higher scores indicate more severe existential distress. In this study, the Cronbach’s α coefficient was 0.812.

### 2.3 Data collection

Paper-based questionnaires were distributed to patients during their hospital stay or dialysis sessions. All data were directly collected by two trained researchers in the hemodialysis center. No other independent personnel were involved during the questionnaire completion process. The researchers received unified training to ensure standardized guidance for patients when filling out the questionnaires. First, eligible patients were informed of the study’s purpose, content, significance, and filling instructions. After obtaining written informed consent, questionnaires were distributed on-site, and patients were asked to complete them based on their actual situation. For illiterate patients, researchers read the questions aloud and recorded answers after confirming them repeatedly with the patients. Finally, questionnaires were collected, numbered, and stored in a locked cabinet to ensure the confidentiality of patients’ personal information. A total of 352 questionnaires were distributed, and 10 invalid questionnaires (with incomplete content or obvious logical errors) were excluded, resulting in 342 valid questionnaires (effective recovery rate: 97.2%).

### 2.4 Statistical analysis

IBM SPSS 23.0 software (IBM Corp., Armonk, NY, USA) was used for data analysis. Normally distributed continuous variables were presented as mean±standard deviation (x̄±s), and categorical variables as frequencies and percentages (*n*, %). Univariate analysis of continuous variables was performed using independent-samples t-tests (for two categories) and one-way analysis of variance (ANOVA) (for three or more categories). Pearson correlation analysis was used to explore the relationships among self-disclosure, intolerance of uncertainty, and existential distress. The PROCESS macro (Model 4) in SPSS was used to test the mediating effect of self-disclosure, with gender, age, and dialysis duration as covariates. A two-tailed *p*-value <0.05 was considered statistically significant.

## 3 Results

### 3.1 General characteristics of participants

Among the 342 participants, 204 (59.6%) were male; the mean age was (52.78 ± 6.52) years, with 165 (48.2%) aged 50 to <60 years; 210 (61.4%) had a monthly family income of 4,000–8,000 RMB; 143 (41.8%) had a dialysis duration of 1–5 years; 296 (86.5%) were married; 167 (48.8%) had a senior high school education; 227 (66.4%) had no fixed job; and 260 (76.0%) received hemodialysis ≥3 times per week.

### 3.2 Scores of self-disclosure, intolerance of uncertainty, and existential distress

The scores of self-disclosure, intolerance of uncertainty, and existential distress in young and middle-aged MHD patients were (38.08 ± 5.97), (45.44 ± 6.10), and (34.39 ± 7.72), respectively. The scores of each dimension are shown in Table 1.

**Table 1:** Scores of self-disclosure, intolerance of uncertainty, and existential distress in young and middle-aged MHD patients (*n* = 342, *x̄*±s, 分).

Variable	Number of items	Score range	Total score	Score rate (%)
Existential distress total	14	0 ~ 56	34.39 ± 7.72	61.4%
Loss of control	3	0 ~ 12	7.69 ± 1.60	64.1%
Loneliness	4	0 ~ 16	10.11 ± 2.31	63.2%
Guilt	3	0 ~ 12	6.92 ± 2.03	57.7%
Meaninglessness/valuelessness	4	0 ~ 16	9.68 ± 3.55	60.5%
Self-disclosure total	12	12 ~ 60	38.08 ± 5.97	63.5%
Intolerance of uncertainty total	12	12 ~ 60	45.44 ± 6.10	75.7%
Prospective anxiety	7	7 ~ 35	26.83 ± 3.68	76.7%
Inhibitory anxiety	5	5 ~ 25	18.61 ± 2.76	74.4%

Score rate (%) = (Actual total score/Maximum possible score) × 100.

### 3.3 Univariate analysis of self-disclosure, intolerance of uncertainty, and existential distress

Univariate analysis showed significant differences in intolerance of uncertainty scores by gender, age group, dialysis duration, marital status, educational level, and employment status (all *p* < 0.05); significant differences in self-disclosure scores by gender, age group, dialysis duration, educational level, and employment status (all *p* < 0.05); and significant differences in existential distress scores by gender, age group, dialysis duration, marital status, educational level, and employment status (all *p* < 0.05) (Table 2).

**Table 2:** Univariate analysis of self-disclosure, intolerance of uncertainty, and existential suffering scores among young and middle-aged patients on maintenance hemodialysis with different characteristics.

Item	Number of cases (%)	Intolerance of uncertainty score	Self-disclosure score	Existential distress score
Gender				
Male	204 (59.6)	46.26 ± 6.80	37.32 ± 6.39	35.07 ± 7.39
Female	138 (40.4)	44.22 ± 4.66	39.21 ± 5.10	33.38 ± 8.10
*t*		3.286	−2.907	2.003
*p*		0.001	0.004	0.046
Age group (years)				
18–<40	45 (13.2)	41.93 ± 6.18	40.11 ± 6.07	31.76 ± 8.36
40 ~ <50	132 (38.6)	45.93 ± 5.65^a^	38.22 ± 5.25^a^	35.36 ± 6.33
50–60	165 (48.2)	46.00 ± 6.14^a^	37.42 ± 6.36^a^	34.33 ± 8.39^a^
*F*		8.960	3.716	3.717
*p*		0.000	0.025	0.025
Average monthly household income (CNY)				
<4,000	106 (31.0)	47.58 ± 4.79	35.38 ± 5.92	37.67 ± 5.38
4,000–8,000	210 (61.4)	44.87 ± 6.41^a^	39.02 ± 5.42^a^	33.42 ± 7.72^a^
>8,000	26 (7.6)	41.27 ± 5.23^a^^,^^b^	41.54 ± 6.46^a^^,^^b^	28.85 ± 10.33^a^^,^^b^
*F*		14.625	19.808	19.925
*p*		0.000	0.000	0.000
Educational attainment				
Junior high school and below	83 (24.3)	41.96 ± 5.33	40.70 ± 4.78	30.69 ± 6.52
High school	167 (48.8)	45.44 ± 5.79^a^	38.40 ± 5.95^a^	33.98 ± 7.78^a^
College and above	92 (26.9)	48.58 ± 5.64^a^^,^^b^	35.15 ± 5.77^a^^,^^b^	38.48 ± 6.29^a^^,^^b^
*F*		29.996	21.637	26.025
*p*		0.000	0.000	0.000
Marital status				
Single (including divorced, widowed)	46 (13.5)	43.39 ± 6.33	39.02 ± 6.25	32.11 ± 6.92
Married	296 (86.5)	45.76 ± 6.01	37.94 ± 5.92	34.74 ± 7.79
*t*		−2.465	1.149	−2.165
*p*		0.014	0.251	0.031
Whether employed				
Yes	115 (33.6)	43.99 ± 5.67	39.09 ± 5.95	32.82 ± 7.99
No	227 (66.4)	46.18 ± 6.19	37.57 ± 5.93	35.19 ± 7.47
*t*		−3.164	2.230	−2.704
*p*		0.002	0.027	0.007
Dialysis duration (years)				
<1	90 (26.3)	48.52 ± 5.14	34.74 ± 5.34	38.98 ± 4.80
1–5	143 (41.8)	45.19 ± 6.06^a^	38.68 ± 6.12^a^	33.66 ± 7.79^a^
>5	109 (31.9)	43.22 ± 5.87^a^^,^^b^	40.06 ± 5.09^a^^,^^b^	31.55 ± 7.94^a^
*F*		21.041	23.488	27.635
*p*		0.000	0.000	0.000
Dialysis frequency (times/week)				
<3	82 (24.0)	45.94 ± 6.44	38.01 ± 5.68	34.76 ± 6.49
≥3	260 (76.0)	45.28 ± 5.99	38.10 ± 6.07	34.27 ± 8.08
		0.852	−0.121	0.493
		0.395	0.904	0.622

a Variable compared with Level 1, *p* < 0.05.

b Variable compared with Level 2, *p* < 0.05.

### 3.4 Correlation analysis of self-disclosure, intolerance of uncertainty, and existential distress in young and middle-aged patients undergoing maintenance hemodialysis

The results of this study showed that in young and middle-aged patients undergoing maintenance hemodialysis (MHD): The score of intolerance of uncertainty was negatively correlated with the score of self-disclosure (*r* = −0.766, *p < 0.01*); The score of intolerance of uncertainty was positively correlated with the score of existential distress (*r* = 0.819, *p < 0.01*); The score of self-disclosure was negatively correlated with the score of existential distress (*r* = −0.817, *p < 0.01*).

### 3.5 Mediation effect analysis of self-disclosure, intolerance of uncertainty, and existential distress in young and middle-aged patients undergoing maintenance hemodialysis

**Figure 1:**
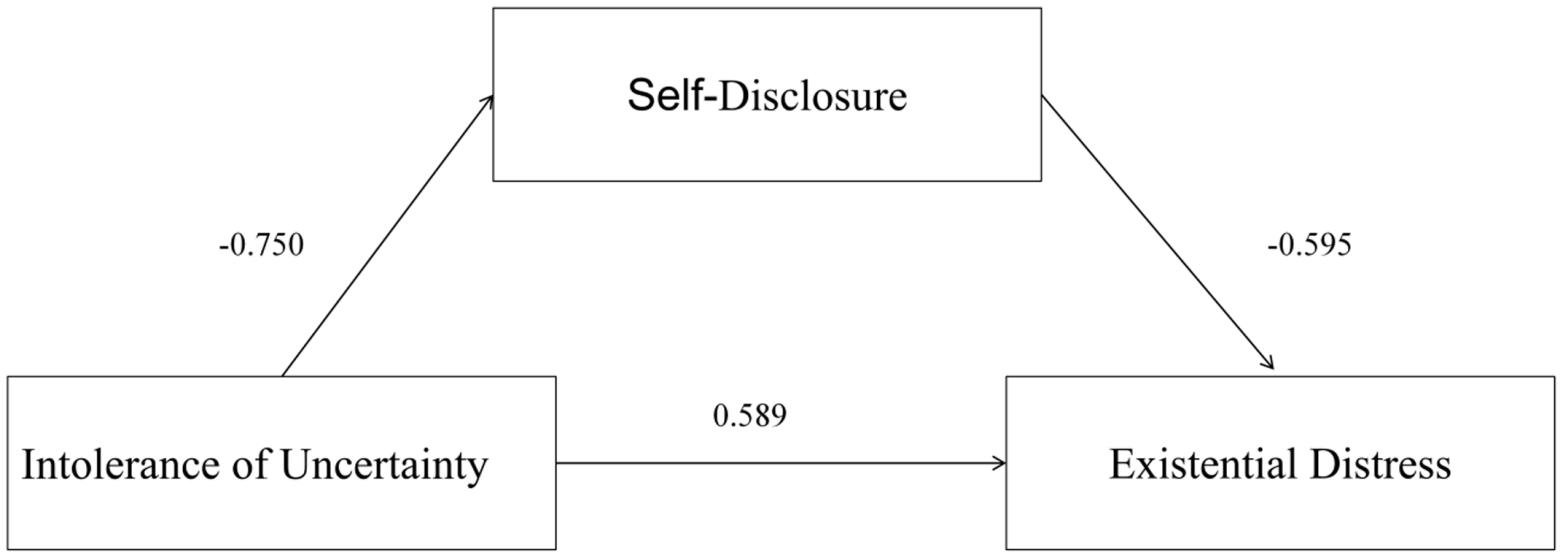
Mediating effect model of self-disclosure.

The PROCESS macro (Model 4) was used for multiple linear regression analysis, with the score of intolerance of uncertainty as the independent variable, the score of self-disclosure as the mediating variable, the score of existential distress as the dependent variable, and the variables with significant differences in univariate analysis as control variables. As shown in Figure 1, the results indicated that:

The score of intolerance of uncertainty had a significant predictive effect on the score of existential distress, with a direct effect of *β* = 0.589 (*p < 0.01*); The score of intolerance of uncertainty could directly and negatively predict the score of self-disclosure, and the score of self-disclosure could negatively predict the score of existential distress; The score of self-disclosure played a partial mediating role between the score of intolerance of uncertainty and the score of existential distress, with a mediating effect of *β* = 0.446 (*p < 0.01*) and a total effect of β = 1.036 (*p < 0.01*).

To ensure the accuracy of this test, the Bootstrap method (Preacher and Hayes, 2008) was employed to conduct 5,000 repeated samples to examine the mediating effect of self-disclosure between intolerance of uncertainty and existential distress. The 95% confidence interval (CI) for the mediating effect of self-disclosure (0.492, 0.687) did not include zero, indicating that self-disclosure mediates the relationship between intolerance of uncertainty and existential distress. This mediating effect accounted for 43.2% of the total effect, thus establishing the mediating model of self-disclosure. See Table 3.

**Table 3:** Analysis of the mediating effect of self-disclosure, intolerance of uncertainty, and existential distress among middle-aged and young patients on maintenance hemodialysis.

Path	Effect size	Standard error	*p*	95% CI	Effect proportion (%)
Total effect	1.036	0.056	0.000	0.925–1.147	100.00
Direct effect: Intolerance of uncertainty → Existential distress	0.589	0.049	0.000	0.492–0.687	56.8
Indirect effect: Intolerance of uncertainty → Self-disclosure → Existential distress	0.446	0.050	0.000	0.357 ~ 0.551	43.2

## 4 Discussion

### 4.1 Current status of self-disclosure, intolerance of uncertainty, and existential distress in young and middle-aged MHD patients

This study found that young and middle-aged patients on maintenance hemodialysis (MHD) experienced moderate to severe levels of existential distress, which was higher than that reported by Yu (2024) in a similar MHD population, indicating a need for targeted interventions. Several factors may explain this finding: (1) The patients in this study were in a critical phase of personal and professional development, bearing significant family responsibilities such as supporting parents and raising children, alongside societal obligations. The sudden onset of illness not only inflicts physical challenges but also profound psychological impacts, predisposing them to negative emotions and heightened existential distress (Hsu et al., 2022). (2) In this cohort, 66.4% of patients lacked stable employment, and 31.0% had a monthly household income of ¥4,000 or less. The requirement for regular weekly hemodialysis directly reduces patients’ working capacity, while disease complications increase treatment costs, exacerbating the financial burden on families. This often leads to feelings of guilt about being a burden, thereby intensifying existential distress.

Gender, age, dialysis vintage, marital status, education level, and employment status were significant factors influencing existential distress scores (all *p* < 0.05). The highest levels of distress were observed in patients who were male, aged 50–<60 years, had a monthly income <¥4,000, were married, unemployed, and had been on dialysis for less than 1 year. This may be attributed to men often bearing greater family responsibilities and experiencing more severe perceived impacts from the disease. Patients aged 50 to <60 face the dual pressure of caring for aging parents while supporting children entering adulthood; the illness often hinders their ability to fulfill these roles, increasing feelings of guilt. Those with low incomes or unstable employment already experience substantial household pressures, which are further amplified by the financial strain of the disease, leading to a heightened sense of helplessness. Patients with less than 1 year of dialysis exposure, being new to treatment, often lack adequate knowledge about dialysis and skills to manage adverse symptoms, potentially fostering fear of the unfamiliar treatment and resulting in more pronounced distress (Xu et al., 2024).

Therefore, healthcare providers should prioritize patients with these characteristics. First, health education should be enhanced, covering dialysis procedures, potential complications, dietary management, physical activity, and preventive measures. This can improve patients’ self-care abilities, help maintain electrolyte balance and nutritional status, reduce multi-organ damage, and ultimately facilitate better adaptation to dialysis for improved survival outcomes. Second, efforts should be made to increase societal support for dialysis patients through policy assistance and psychological aid, enhancing their self-efficacy and alleviating the disease burden. Finally, healthcare teams should actively foster patients’ internal resilience, accurately identify negative psychological states, and promote positive psychological adaptation through mindfulness training, patient education, and peer support programs, enabling patients to achieve an optimal state of adjustment to their condition.

The self-disclosure score in this population was 38.08 ± 5.97, indicating a moderate level, which was higher than that reported by Qiu et al. (2024) (Chinese hemodialysis patients) but lower than that of chronic disease patients with other diseases (e.g., gynecological malignant tumors; Liu et al., 2025) possibly due to the unique body image changes and social stigma of MHD patients. End-stage renal disease, with its irreversible kidney damage, leads to complications such as anuria, characteristic facial changes, and oral odor, which can foster negative psychological states. The perceived stigma associated with the disease may further discourage patients from discussing their condition with others. Additionally, hemodialysis requires stable vascular access, and repeated needle insertions can cause visible changes to the arms. Such disturbances in body image often lead patients to withdraw from social contacts, resulting in significant social isolation and consequently lower levels of self-disclosure (Sharif Nia et al., 2022). This suggests that healthcare providers should pay close attention to patients with low self-disclosure. Regularly organizing peer support groups can provide a platform for patients to share experiences and feelings, thereby expanding their avenues for emotional expression. Furthermore, a comprehensive management approach is essential, encompassing not only disease-specific education but also psychological care. Patients should be encouraged to engage in positive emotional expression through methods like journaling, benefit-finding, and self-acceptance to alleviate negative emotions and promote overall well-being.

The intolerance of uncertainty score was 45.44 ± 6.10, indicating a moderately high level, which exceeds findings from Yi et al.’s (2023) study on colorectal cancer patients. As dialysis continues, patients may experience complications like electrolyte imbalances, anemia, and heart failure. Simultaneously, strict fluid and dietary restrictions necessitate significant lifestyle changes. Facing these potential uncertainties places a substantial psychological burden on patients, reducing their tolerance for ambiguity. Furthermore, the time-consuming and regimented nature of dialysis treatment can foster feelings of helplessness about the future, diminishing hope and increasing intolerance of uncertainty. For patients with high intolerance of uncertainty, healthcare providers should implement early interventions. This includes proactively providing patients and their families with knowledge and skills for post-dialysis health management, emphasizing that adherence to treatment and strict self-management can significantly improve quality of life and enable a successful return to daily activities and work. Implementing holistic, life-cycle health management services can offer convenient and professional consultation and follow-up care, helping to identify and address emerging patient crises promptly, thereby reducing uncertainty, restoring hope, and improving quality of life.

### 4.2 Correlations among self-disclosure, intolerance of uncertainty, and existential distress

A positive correlation was found between intolerance of uncertainty and existential distress (*r* > 0, *p* < 0.01), suggesting that higher intolerance of uncertainty is associated with more severe existential distress. On one hand, the complexity and unpredictability of the disease and its treatment can lead to persistent feelings of uncertainty about the future, which directly negatively impacts emotional state, potentially manifesting as anxiety or depression, thereby increasing existential distress (Qiu et al., 2024). On the other hand, the regular dialysis regimen often results in a partial loss of working capacity, and the combined economic, physical, and psychological strains directly contribute to heightened distress.

Self-disclosure was negatively correlated with existential distress (*r* < 0, *p* < 0.01). This result is consistent with the findings of Yuan (2025) in patients with head and neck cancer, indicating that better self-disclosure is associated with lower levels of existential distress. Patients with higher self-disclosure tend to develop more effective coping strategies. By articulating their emotions, they can better process their feelings and actively seek external support, thereby enhancing their resilience to stress. This adaptive coping style directly mitigates feelings of existential distress and supports psychological and physical recovery.

### 4.3 The mediating role of self-disclosure between intolerance of uncertainty and existential distress

Self-disclosure played a partial mediating role between intolerance of uncertainty and existential distress, accounting for 43.2% of the total effect. This indicates that intolerance of uncertainty influences existential distress both directly and indirectly through its negative impact on self-disclosure.

This mediating effect can be explained by Uncertainty Reduction Theory (Yang et al., 2020; Lu et al., 2024), and is consistent with the findings of a self-disclosure mediation study among peritoneal dialysis patients in China (Ning, 2024), confirming that self-disclosure is a key mediating variable in the psychological pathway of MHD patients. Individuals often seek information through social interaction to increase predictability and understanding of uncertain situations. Patients with a greater tendency for self-disclosure are more likely to utilize available resources to express themselves and confide in others, gaining valuable support and information. This improved understanding of their condition and treatment fosters proactive coping, thereby reducing the negative psychological impact of illness and lessening existential distress (Lu et al., 2025). Furthermore, effective self-disclosure can facilitate cognitive restructuring – allowing patients to reframe and reinterpret the uncertainties associated with their treatment and recovery. By expressing their thoughts and feelings, they can gain new perspectives, enhance hope, cultivate a more positive mindset, and consequently reduce distress.

These findings suggest that healthcare providers should focus on patients exhibiting high intolerance of uncertainty. Interventions should aim to improve patients’ disease knowledge and self-management skills to encourage active treatment engagement, thereby reducing anxiety. Additionally, structured group interventions designed to foster self-disclosure can be beneficial. These should address dimensions such as illness perception, emotional expression, cognitive reframing, and resource acquisition to help patients effectively articulate their feelings and values, alleviate psychological burdens, reduce existential distress, and improve overall outcomes (Li R. et al., 2024; Li X. et al., 2024; Wang X. et al., 2024; Wang Y. Y. et al., 2024).

## 5 Conclusion

In summary, this study found that young and middle-aged MHD patients experience moderate to severe existential distress, warranting attention from healthcare professionals and families. Self-disclosure partially mediates the relationship between intolerance of uncertainty and existential distress. This suggests that: (1) Healthcare systems should establish routine communication channels to reduce patient uncertainty through information transparency and peer support initiatives; (2) Nursing teams can utilize cognitive-behavioral techniques and group therapy to guide self-disclosure and educate families to create supportive communication environments; (3) A collaborative “medical + psychological + family” care model should be developed, involving dedicated psychological nurses to assess distress and train families in supportive strategies. This integrated approach can reduce uncertainty, foster positive psychology, mitigate existential distress, and enhance quality of life.

## 6 Limitations

This study has several limitations. (1) The participants were recruited from a single tertiary hospital, limiting the geographical and cultural diversity of the sample and potentially affecting the generalizability of the findings. (2) The cross-sectional design precludes definitive causal inferences between the variables. Future research should involve multi-center studies with larger, more diverse samples to explore potential cultural and regional variations. (3) Data were collected during dialysis sessions. Although this ensured accessibility, patients’ responses might have been influenced by treatment-related discomfort or fatigue, which could potentially bias the reporting of psychological states. Based on these findings and limitations, future research should: (1) employ longitudinal designs to establish causal relationships among intolerance of uncertainty, self-disclosure, and existential distress over time; (2) recruit culturally and geographically diverse samples from multiple centers to improve generalizability; and (3) develop and test targeted interventions (e.g., uncertainty management training, facilitated self-disclosure groups) to evaluate their efficacy in reducing existential distress in young and middle-aged MHD patients.

## Data Availability

The original contributions presented in the study are included in the article/supplementary material, further inquiries can be directed to the corresponding author.
